# When Is Being Sad on the Burn Unit Pathological? Differential Diagnosis of Demoralization, Adjustment Disorder and Major Depressive Disorder in Burn Survivors

**DOI:** 10.3390/ebj3010010

**Published:** 2022-02-10

**Authors:** Marissa L. Beal, Sheera F. Lerman, Idris E. Leppla

**Affiliations:** Department of Psychiatry and Behavioral Sciences, Johns Hopkins University School of Medicine, Baltimore, MD 21224, USA; szohar1@jhmi.edu (S.F.L.); ileppla1@jhmi.edu (I.E.L.)

**Keywords:** demoralization, adjustment disorder, major depressive disorder, burns

## Abstract

Many burn survivors have pre-existing psychiatric conditions or develop psychological or psychiatric symptoms over the course of their hospital stay. Patients often present with low mood and neurovegetative symptoms which can be conceptualized as demoralization, adjustment disorder, or major depressive disorder. We review the literature on these syndromes in burn survivors and present three cases that highlight the continuum of these syndromes for patients who present with symptoms of depression following a burn injury. We discuss the clinical challenges of differentiating these syndromes as well as psychotherapeutic and psychopharmacologic considerations and recommendations.

## 1. Introduction

It has long been known that there are adverse psychological consequences in burn survivors [1]. Many burn survivors have pre-existing psychiatric conditions [2] or develop a psychiatric condition or psychological distress following their injury [3]. During acute hospitalization, the psychiatrist or psychologist is often called to the bedside for a patient following a burn injury for low mood, hopelessness, poor appetite, or decreased motivation to participate in care. While often these symptoms are recognized by the primary team as depression, the severity of symptoms can vary for each patient. When presenting in the setting of an acute stressor such as a burn injury, there is a continuum of low mood, ranging from a normal response to adversity to an exaggerated response to a stressor to a complete disturbance in someone’s mood and energy [1,4]. This range of affective change can be conceptualized as either demoralization, adjustment disorder, or major depressive disorder (MDD). Demoralization, which describes the mildest symptoms on this continuum, is a syndrome characterized by symptoms of low mood that are understandable in the context of the stressor [5]. Demoralization is not currently a DSM-V diagnosis but is a syndrome recognized in the literature that may characterize the psychological response of some burn survivors following their injury and treatment. While these terms are often used interchangeably, it is vital to appropriately identify and treat the patient based on where they may fall on this continuum. This paper presents three patient cases to help highlight and differentiate these syndromes and reviews management strategies for patients with psychological and psychiatric sequelae in burn survivors.

## 2. Defining the Syndromes

### 2.1. Demoralization

Demoralization, as described by Slavney [5], refers to a normal and understandable response to a certain stressful circumstance. With the most extreme circumstances, demoralized people can have severe symptoms, such as grief, hopelessness, feelings of isolation, and fear. Even the most psychologically resilient individual might experience demoralization in the face of an extreme stressor. The determination of what constitutes a reasonable or appropriate psychological response to a stressor is in the subjective eyes of the clinician, which can leave room for inconsistencies in identification. Jerome Frank defined demoralization as an inability to handle stress, including feelings of despair, isolation, and low self-esteem, which often lead people to seek psychotherapy [6]. Frank argued that supportive psychotherapy and “remoralizing” the patient by reframing their difficulties could have a profound impact on the trajectory of a patient’s course.

As stated previously, demoralization is yet to be an official psychiatric diagnosis defined in the DSM-V; however, it can have symptoms similar to depression, such as hopelessness and low mood. It was also defined by de Figuieredo [7] as a combination of distress and subjective incompetence that is “exogenomorphic” or in relation to a failure or stress that is external. He describes subjective incompetence as a perceived inability to behave in an appropriate way following a stressor [8]. He later went on to propose diagnostic criteria for demoralization, including the presence of subjective incompetence, helplessness, hopelessness, and presence of distress which take place in the setting of stress and causes significant impairment in functioning [9]. Kissane and colleagues [10] defined the demoralization syndrome as existential distress, hopelessness, helplessness, and meaninglessness. Clarke and Kissane [11] argued that demoralization falls not on a continuum with depression but is its own entity that can be comorbid with other psychiatric illnesses such as MDD or schizophrenia. Demoralization is also discussed in the literature with various terminology, including psychological distress [12], Giving-up–Given-up syndrome [13], and suffering [14].

Though these definitions vary, demoralization is an entity frequently encountered in hospitalized patients and one that should be recognized in burn survivors. For these patients, the burn injury itself is a trauma; however, prolonged hospitalization, painful interventions, and repeated surgeries can also constitute stressful circumstances leading to demoralization.

#### Case 1: Demoralization

*A 53-year-old woman was admitted to the burn unit after a 1% TBSA burn to her foot after soaking it in boiling hot water, which she did not notice due to sensory deficits. Psychiatry was consulted because the patient had a history of depression and appeared sad in the interview. She also reported trouble sleeping in the hospital. In the interview, the patient talked favorably about her vocational history and was frustrated by her sensory deficits and immobility. She reminisced about working full time, lamented that she was no longer able to chase her grandchildren around the house, and felt “inept” as a grandmother. She sometimes felt like a burden to her family, especially because they had to help her around the house due to difficulties with ambulation, now exacerbated in the setting of an additional injury. She was participating in physical and occupational therapy.*

This case represents demoralization as this is a patient with a chronic medical condition with a worsening mood in the setting of a burn injury. Even prior to her burn injury, she was frustrated about her current state in life and wished to return to her prior level of functioning, indicating symptoms of demoralization which increased during her hospitalization. She noted low mood, trouble sleeping, and guilt about burdening her family due to functional deficits. While mood symptoms were present, they appeared to be in proportion to the stressor, and she was participatory in her care. Her mood also improved when she talked about her prior accomplishments, which is a sign that her mood is reactive, as opposed to overtly negative. She also has a history of depression and thus is at risk for a subsequent depressive episode should her symptoms worsen.

### 2.2. Adjustment Disorder

Adjustment disorder has similar symptoms as demoralization; however, it is pathologized as a psychiatric disorder in the DSM-V. Pathologized, in this context, refers to an individual who is adjusting poorly to his/her new set of circumstances. Adjustment disorder is defined as “the development of emotional or behavioral symptoms in response to an identifiable stressor(s) occurring within 3 months of the onset of the stressor(s)” [15]. These symptoms need to cause (1) marked distress out of proportion to the severity or intensity of stressor or (2) significant impairment in social, occupational, or other important areas of functioning. As with demoralization, this diagnosis requires the clinician to determine if the response is out of proportion to the severity of the stressor and may be clinician dependent. For example, some clinicians might find that daily tearfulness and low mood is expected if someone loses an arm due to an electrical injury. Other clinicians might expect that the patient would have a period of distress but ultimately harness internal coping skills to avoid daily tearfulness. Some clinicians diagnose adjustment disorder if there is a low mood in addition to a significant stressor, whether or not the response seems exaggerated. Since there is clinician subjectivity in these diagnoses, it makes sense that there is not a uniform formulation of the definition of an adjustment disorder.

In the classic definition of adjustment disorder, symptoms should develop within 3 months of the onset of the stressor, and symptoms should resolve over time (6 months) as the stressor resolves. This time frame is often not applicable to burn survivors given their prolonged recovery and rehabilitation process, which can span across months and years. Additionally, changes to appearance and function as well as each intervention burn survivors might undergo, such as wound care or reconstructive surgery, can be seen as a new stressor triggering a negative emotional response. The DSM-V accounts for this by saying, “If the stressor or its consequences persist, the adjustment disorder may also continue to be present and become the persistent form” [15]. Many burn patients will develop persistent adjustment disorder as recovery from initial injury often requires more than 6 months, or the injury itself can change the trajectory of someone’s life. Adjustment disorder is a common diagnosis for patients who are medically ill [16]. However, research is limited in this area due to the lack of standardized measurements and variability in diagnostic criteria between ICD-10 and DSM-V. “In a hospital psychiatric consultation setting, adjustment disorder is the most common diagnosis, frequently reaching 50%” [15]. In ICD-10, adjustment disorder is defined as a preoccupation with a stressor and failure to adapt to new circumstances, interfering with everyday functioning [17]. The ICD-10 definition also includes symptoms characteristic of traumatic disorders, such as irritability, trouble sleeping and flashbacks. Notably, adjustment disorder does not meet the criteria for another mental disorder and is not an exacerbation of a previously existing mental disorder [15]. Patients who meet the criteria for MDD would then be diagnosed with MDD and not adjustment disorder in both the ICD and DSM definitions. DSM-V also states that symptoms do not represent normal bereavement but does not mention or exclude demoralization [15].

#### Case 2: Adjustment Disorder

*A 25-year-old woman presented with a 3rd degree 1% TBSA scald burn to her index finger after accidentally pouring boiling water on her hand. Prior to her hospital admission, the patient was taking care of her children and working part-time. During the course of her hospitalization, she had multiple crying spells and told providers that she was “struggling to cope with life.” She declined calls from her children. She also seemed to require much more pain medication than other patients with similar injuries. She did not require surgery but was unwilling to participate in dressing changes and seemed unmotivated for discharge.*

This patient notably reported low mood, difficulty coping, poor sleep, and increased irritability following her injury. The staff recognized that her symptoms and pain seemed out of proportion to the injury and her symptoms impacted her functioning. She does not yet meet the criteria for MDD, and since her symptoms are in the context of a stressor, specifically her burn injury, she meets the criteria for a diagnosis of adjustment disorder. That being said, adjustment disorder can morph into a syndromic MDD if not treated aggressively. In one study of physical trauma survivors, participants who were diagnosed as having an adjustment disorder at 3 months were more likely to meet the criteria for an Axis I disorder at 12 months, indicating that an adjustment disorder diagnosis may be a harbinger of more serious psychiatric pathology later on [18].

### 2.3. Major Depressive Disorder

MDD, as defined in the DSM-V, identifies symptoms lasting for at least 2 weeks with at least five out of nine symptoms, consisting of at least one of two primary symptoms, including depressed mood or anhedonia. Other symptoms include a change in sleep, change in appetite, psychomotor agitation or retardation, low energy or fatigue, feelings of worthlessness or excessive guilt, decreased ability to concentrate, and thoughts of death or suicidal ideation. For patients who are medically ill, the illness itself may lead to low appetite and fatigue, which confuses the diagnosis of MDD further. Though the DSM-V does indicate that symptoms must not be due to a medical condition [15], this is often difficult for practitioners to distinguish. There are different approaches to diagnosing MDD in the medically ill, such as inclusive, exclusive, substitutive, and etiologic. The exclusive approach does not count symptoms of anorexia and fatigue and only requires four out of seven symptoms for a diagnosis of depression [19]. The inclusive approach counts all symptoms and does not distinguish between the origin of symptoms. The substitutive approach substitutes the neurovegetative symptoms of depression, which may be attributed to a medical condition, for psychological symptoms, including fearfulness, social withdrawal, and self-pity [20]. The etiologic approach, as described by the DSM-V, requires clinicians to determine if the symptoms are due to an underlying medical condition and thus would not be counted towards a diagnosis of MDD, making this approach clinician dependent [15]. As the inclusive approach is the most sensitive, some argue this is the best approach clinically as depression is often underdiagnosed in patients who are medically ill [21] and specifically burn patients as it is difficult to distinguish the etiology of symptoms [22].

#### Case 3: Major Depressive Disorder

*A 46-year-old woman with a history of recurrent MDD is admitted due to 20% TBSA to her abdomen due to self-inflicted burns. The patient had a history of psychiatric hospitalizations and appeared unmotivated during her recovery. She was minimally engaged with staff and required a nasogastric tube due to lack of oral intake. Her mood was “numb”, and she slept off-and-on during the day, although she appeared attentive and alert when interviewed. Her family attempted to cheer her up with daily visits, but her mood did not seem to brighten. She told the psychiatric consultant that she was upset that her plan for suicide did not work.*

For case 3, the patient had a history of MDD and thus was at risk for another episode in the setting of her burn injury and hospitalization. However, unlike case 1, the patient had six symptoms of depression: low mood, anhedonia, low motivation, poor appetite, poor sleep, and suicidal ideation, and so even though a stressor was present, she met the criteria for MDD.

### 2.4. Acute Stress Disorder and Post-Traumatic Stress Disorder (PTSD)

Though not the focus of this review, when thinking of this continuum, we must also mention acute stress disorder and PTSD. These diagnoses are prevalent in burn survivors [23] and should remain on the differential when evaluating patients. Likely in the acute care setting, clinicians should first consider acute stress disorder as these symptoms occur within 1 month of the stressor, though notably, some burn patients may have hospitalizations longer than one month. Acute stress disorder has some symptoms that overlap with demoralization, adjustment disorder, and MDD, including negative mood as well as alterations in arousal and reactivity (irritability, problems with concentration, sleep disturbance) [15]. However, to meet the criteria for acute stress disorder or PTSD, the presence of intrusive symptoms (such as nightmares, flashbacks, intrusive memories), avoidance of stimuli, or dissociative symptoms must also be present. During the initial evaluation, intrusive, avoidance, dissociative, and arousal (specifically hypervigilance, exaggerated startle reflex, and anger outbursts) symptoms should also be screened for as these symptoms may not be elicited when screening for MDD.

## 3. Distinguishing between the Three

Distinguishing between these syndromes is difficult, though some key differences are outlined in Table 1. De Figueiredo [7] identified a distinction between depression and demoralization, including the source of distress being external in demoralization versus internal with depression. He also highlighted that while both appear unmotivated, in MDD, patients are unmotivated even when they know what should be done. In demoralization, decreased motivation presents as indecision, with the course of action defined as subjective incompetence [8]. Anhedonia can also be used to distinguish depression from demoralization, as anhedonia is more often associated with MDD [24]. Demoralized people are able to experience joy so long as external circumstances allow, but their circumstances often limit the amount of joy they are able to experience. In case 1, we see a woman who was coping with a change in life circumstances following her injury. While her mood symptoms did not seem severe enough to impact her functioning, she still struggled with frustration, subjective incompetence, and a change to her vision of “self” with regard to her immobility.

In burn survivors, it is even more challenging to differentiate these diagnoses. Since neurovegetative symptoms are frequently seen in burn survivors [25,26], at times, it is unclear which is a symptom of depression or secondary to sequelae of a burn or treatment of a burn. Furthermore, all burn survivors experience an acute stressor of the burn injury and often prolonged hospitalization, multiple treatments, and physical changes, including scarring and contractures. Clinicians must then determine if affective symptoms are in proportion or out of proportion to the stressor to distinguish between demoralization and adjustment disorder, which is subjective and clinician dependent. Casey [27] highlighted variables to consider when differentiating between adjustment disorder and normal response to a stressor, including personal circumstances and the context of the stressor, proportionality between symptoms severity and the triggering event, the persistence of symptoms beyond the expected timeframe, cultural norms, and the duration and severity of functional impairment. This is highlighted when reflecting on the patient depicted in case 2, who had minimal injuries, yet her reaction and subsequent emotions were much greater than the stressor and lasted much longer than expected.

The patient in case 3 had significant anhedonia and history of MDD. Her mood was not reactive and was persistently low. She had ongoing passive death wishes. Her mood was presumably low prior to her burn as the burn was self-inflicted therefore her mood does not appear to be a result of a stressor. In any patient with a prolonged hospital course with a period of intubation, hypoactive delirium should be ruled out [28], especially as highlighted in case 3, since she had disordered sleep. Hypoactive delirium can also include symptoms of low mood, poor sleep, and poor concentration, so a cognitive assessment such as MMSE or MOCA is recommended to further elucidate the etiology of symptoms and determine if an underlying delirium is present. In this patient, given her clarity of ongoing passive death wishes and intact attention, she does not appear to be delirious.

## 4. Prevalence of These Syndromes in Burn Survivors

Depressive symptoms and diagnoses are prevalent in burn survivors, but demoralization is not a term frequently used in the literature to identify this syndrome. While the prevalence of demoralization is unknown in burn survivors, psychological distress is frequently documented and discussed without a common language for all clinical providers to use [4]. There is a subset of patients who develop psychological distress that is identified during hospitalization or rehabilitation, but symptoms are not severe enough to warrant a DSM-V diagnosis, such as MDD [1]. Often these patients do exhibit higher rates of psychological distress greater than the average population, and these symptoms endure over time [4,29]. Patients who develop psychological distress while hospitalized also had a slower rate of recovery [30]. This psychological distress may be indicative of a diagnosis of demoralization and could be helpful for members of a multidisciplinary burn team who see patients with psychological distress to have the terminology to identify, communicate, and treat these symptoms.

While there is limited data regarding the prevalence of adjustment disorder in burn survivors specifically, there is some data for the prevalence and outcomes following a trauma injury. In a longitudinal study of trauma survivors at 3 and 12 months post-injury (most common injury was MVC followed by a fall), 19% of patients had adjustment disorder at 3 months and 16% at 12 months [18]. Symptom severity (loss of sleep, irritability, anxiety) for patients with adjustment disorders were worse than for patients with no psychiatric disorder but better than patients with another primary psychiatric disorder such as MDD or panic disorder [18].

The prevalence of MDD in burn survivors in the hospital and 1 year following burn ranged from 4–10% when using a structured interview and between 4–54% when using self-reported questionnaires [31]. The prevalence rates are varied due to a lack of standardized methods of measurement in the literature. Burn survivors are more likely to have pre-existing psychiatric disorders and worse outcomes after their injury [32]. Burn survivors with pre-existing mood or anxiety disorders had worse social and emotional outcomes at discharge compared to patients without, but these differences were not sustained over time after discharge [33]. Those with pre-existing psychiatric conditions also had a prolonged hospital length of stay following a burn injury [2], worse hospital course outcomes, and disposition barriers [34].

## 5. Treatment

The treatment options for these three syndromes may overlap, as outlined in Table 2. Specifically, psychotherapy is indicated for all patients, and there is limited evidence on which modality is most efficacious for burn survivors. Pharmacotherapy is not indicated for patients with demoralization unless symptom severity worsens or begins to impact functioning and then likely would meet criteria for an adjustment disorder.

### 5.1. Psychotherapy for Burn Patients with Depressive Symptoms

The traumatic nature of a burn injury combined with changes in appearance, function and experience of pain often leads to significant psychological distress for burn survivors [4]. Evidence for the specific treatment of adjustment disorder or MDD in this population is limited, yet a number of studies demonstrated positive outcomes in reducing burn survivors’ depressive symptoms in both the inpatient and outpatient settings [35,36]. However, due to the use of symptom severity questionnaires rather than specific psychiatric diagnoses in these studies, it is not possible to conclude if psychotherapy is more appropriate for a specific depressive disorder.

When considering psychotherapy for patients with demoralization, it is important to recognize that these patients may have trouble making sense of their new set of circumstances. A primary goal of bedside psychotherapy is to imbue the patient with a new sense of purpose and identity [37]. The patient in case 1 had thought of herself primarily as an active worker. Now that her mobility is impaired, she does not have the daily sense of accomplishment that she was accustomed to. She is now struggling to adapt to her identity as someone who needs help with basic tasks. One goal of bedside psychotherapy is to reassure the patient that her feelings are normal and that many people would feel a sense of loss in these circumstances. Sometimes the best treatment for demoralization is to provide psychoeducation to both the patient and the team about the non-pathologic response of suffering, including the fact that it is acceptable to have periods of sadness and frustration in response to difficult circumstances [37].

For adjustment disorders, psychotherapy, mostly CBT type interventions, are considered the first line of treatment and were found to be effective in reducing symptoms of depression and anxiety [38,39]. However, there continue to be significant gaps in the literature for the diagnosis and treatment of adjustment disorders [40] and a need for quality RCTs to determine the effectiveness of different interventions for medically ill patients in general [41] and for burn survivors specifically. While psychotherapeutic interventions for depression such as CBT, interpersonal therapy, and problem-solving therapy have all shown large treatment effects compared to waitlist [42], to the best of our knowledge, there are no studies assessing these interventions in burn survivors. Hence, there is not sufficient evidence to conclude if different treatment considerations are required for those with comorbid medical conditions [43], such as burn injuries.

In their excellent review of the literature, Rosenblat and colleagues [44] propose a Stepped Care model for the treatment of depression in medically ill patients, based on the severity of their depressive symptoms. Psychotherapeutic interventions are recommended for all levels of severity, with increasing intensity of psychotherapy and combination with pharmacotherapy as symptoms become more severe. It is likely that this treatment model would be applicable for burn survivors, though further research is required to determine the need for burn specific modification.

An important treatment consideration is that access to psychotherapy for burn survivors is often limited, given the shortage of mental health providers who can deliver these interventions in both the inpatient and outpatient settings [45]. Medication, however, is more readily available and can be prescribed by the primary medical team or consulting psychiatry service. Interestingly, one study demonstrated that early pharmacological interventions for adjustment disorder were associated with increased risk for later psychiatric hospitalization, while early psychotherapy was associated with reduced risk [46], highlighting the importance of appropriate treatment choices for each disorder.

### 5.2. Psychopharmacology Considerations for Burn Patients

Before starting psychopharmacology for either adjustment disorder or MDD, it is important to first rule out medical causes of depression, including CNS pathology (such as stroke or tumor), endocrine disorders, infections, vitamin deficiencies, and/or substance-induced withdrawal (typically from opioids/benzodiazepines) [47,48]. The psychiatric consultant and primary team should also ensure a complete medication check is completed during the admission to ensure any psychiatric medications from prior to admission are restarted and continued if appropriate. If an antidepressant was held during an acute injury, it should likely be restarted when the patient is lucid and able to converse. When continuing or starting new psychotropic medications, it is important to think about alterations in metabolism and drug–drug interactions as the patient is now in a critical care setting, likely with a new extensive list of medications. It is important to consider interactions with other medications and variable hepatic or renal function, which may impact drug metabolism. Treatment teams should be aware of the risk of serotonin syndrome as serotonergic agents may be added during the hospitalization, such as antiemetics and opioids [49]. It is also important to consider the additive effect of sedating medications when burn survivors are still in critical care settings [50]. That being said, it is vital not to undertreat depressive symptoms, and it is important to avoid delaying restarting home psychotropics.

It is also important to identify and treat pain as depressive symptoms and pain are often comorbid [51]. Though there are numerous challenges associated with the treatment of pain in burn patients, the psychiatrist and psychologist should continue to evaluate and discuss multimodal pain management options with the multidisciplinary team to prevent exacerbation of mood symptoms [52].

#### 5.2.1. Psychopharmacology: Adjustment Disorder

Psychopharmacology for the treatment of adjustment disorder includes symptomatic treatment though there is limited data regarding appropriate psychopharmacology and none regarding the treatment of adjustment disorder in burn survivors. For symptomatic treatment, patients with sleep disturbances are prescribed a sleep aid such as mirtazapine or trazodone. Patients with a lack of appetite may need an appetite stimulant such as mirtazapine or olanzapine. Those with anxiety, panic attacks, or insomnia may benefit from a benzodiazepine [27]. However, it should be noted that there is a high rate of substance use disorder in burn survivors, and thus a history of substance use should be evaluated prior to initiation of a benzodiazepine [53]. Adjustment disorder is also now considered a trauma/stress-related disorder, and benzodiazepines are not recommended for others in this category, such as PTSD and acute stress disorder [54]. There are few trials indicating the efficacy of antidepressants for adjustment disorder, though one retrospective case study showed higher efficacy for SSRIs for the treatment of adjustment disorder compared to MDD [55]. Another study showed no benefit of psychopharmacology when compared to placebo or psychotherapy [56]. Two studies looked at the efficacy of extracts for the treatment of adjustment disorder with anxious mood, specifically kava kava and Valerian root, compared to placebo and saw a greater improvement in symptoms with extracts [57,58]. With limited data in the burn population, we recommend using symptomatic pharmacologic treatment to target symptoms that may be impacting care and progress in rehabilitation.

#### 5.2.2. Psychopharmacology and Neuromodulation: MDD

For burn patients who meet the criteria for MDD, there is limited data regarding the most efficacious psychopharmacologic treatment for this patient population, and thus we must rely on treatment recommendations for the general population and those with other medical comorbidities [44]. The first-line treatment for MDD in medically ill patients is SSRIs as they are generally well-tolerated, though SNRIs, bupropion, and mirtazapine are all appropriate first medication trials for patients with MDD [59]. It may be useful to think about comorbid symptoms for burn survivors and utilize psychopharmacology to target these symptoms, as discussed for adjustment disorders. For example, SNRIs, such as venlafaxine and duloxetine, or TCAs, may be helpful for the treatment of pain as well as MDD [60]. Patients who do not respond to an initial trial of medication may then need to be switched to a different medication either in the same class or a different class. Since burn survivors often have prolonged hospital stays, this may be indicated in the acute setting. While an appropriate trial of the medication at an adequate dose is recommended, if noted to be ineffective, switching to a different class of medications may be warranted.

Electroconvulsive therapy (ECT) may also be appropriate for patients who are treatment-resistant and have refractory or severe symptoms. ECT has been utilized in other medically ill patient populations, including those with cancer, Parkinson’s disease, and dementia and demonstrated efficacy and tolerability [61,62,63]. There are a few case reports of patients with burn injuries requiring ECT for treatment of mood or psychotic symptoms following a burn, with one case actually receiving ECT on the critical care unit [64,65,66]. ECT is used so rarely for burn survivors because the typical anesthetic used is succinylcholine, and this can lead to hyperkalemia in burn patients [67]. Other anesthetics are used, so this should not entirely exclude the use of ECT for this patient population.

Repetitive transcranial magnetic stimulation (rTMS) has increasing evidence for medically ill patient populations, such as patients with Parkinson’s disease or a history of stroke [68,69]. rTMS does not require anesthesia or sedation, so it may be safer for burn patients than ECT. Although, the accessibility of rTMS for patients who are hospitalized or in rehabilitation facilities may be limited.

## 6. Discussion

There are many challenges in regard to identifying and treating these syndromes. For one, there is a great deal of overlap in symptomatology between the three, making it challenging and often subjective to differentiate between them. The burn injury itself is an acute stressor for most survivors, with some enduring unimaginable and intense trauma making it challenging for the clinician to quantify whether the patient’s emotional reaction is appropriate or pathological. Time and experience working with burn survivors may assist the clinician in the conceptualization of a “typical” or “expected” clinical course of emotional responses to burn injuries and aid in differentiating between adaptive and maladaptive adjustment. The clinician should also rely on the interdisciplinary team to identify when emotional reactions or responses seem out of proportion to the stressor, and information from nursing, occupational and physical therapists, wound care nurses, and surgeons will help to provide the clinical context necessary for the psychiatrist/psychologist to make an appropriate assessment.

While difficult, it is important for both the provider and patient to distinguish these syndromes. As outlined above, each syndrome requires a different management strategy. With demoralization, the management is often psychoeducation to both the patient and the treating clinicians [5]. Slavney [5] noted the discomfort of the treating physician to witness psychological distress, but also the relief of the patient to understand their symptoms are not pathological or due to a psychiatric condition.

The next consideration is the clinical utility of distinguishing these three syndromes in practice as well as challenges with billing and reimbursement. In the United States, most mental health providers will use mental and behavioral disorders codes (namely F codes) for billing of psychiatric assessment and intervention. While adjustment disorders and MDD are categorized under these mental health codes, demoralization is not (ICD R45.3; symptom code), making it more difficult to associate this diagnosis with clinical encounters and receive reimbursement. Slavney [5] recommended utilizing an ICD V code for reimbursement, but we recognize that most clinicians will likely use an F code and thus code demoralization as an adjustment disorder, partially for the ease and simplicity of billing. Often, psychiatrists are consulted when symptoms are impacting care or significant enough to impair functioning in the hospital setting. One could make an argument that if the team feels that a consult is warranted, then the symptoms are now above the level of a normal response to a stressor and, therefore, at least warrants a diagnosis of adjustment disorder. However, with the increased emphasis on multidisciplinary care and proactive consults, this may not always be the case. Psychologists and psychiatrists may be engaged in care even before symptoms begin impacting functioning and thus may evaluate patients who are at the beginning of the continuum and only demoralized. It may be helpful to signal to the clinician and other providers that demoralization is present with an R code, indicating the syndrome of demoralization even if adjustment disorder is billed as well.

Another issue is that one patient may experience all three syndromes during the span of his/her illness. For example, our patient in case 3 could have initially reported sleeplessness, irritability, and hypervigilance during the early course of her hospitalization, leading to a diagnosis of adjustment disorder, necessitating treatment with a sleep aid and relaxation techniques. Then, after prolonged hospitalization, the patient starts to show symptoms of MDD characterized by anhedonia, hopelessness, and suicidal ideation. The patient may need specialized psychiatric evaluation, a robust trial of an antidepressant, and weekly psychotherapy. After several years into the patient’s course, the patient is no longer depressed or anxious but still experiences existential angst and anger about this injury and thus faces constant demoralization, similar to the experience outlined in case 1. Thus it is important to continue to screen and re-evaluate patients as they progress through their medical course.

While we focused this manuscript on the identification and treatment in the acute care setting, a burn survivor’s clinical course extends far beyond their time as an inpatient in the hospital. These patients often require continued rehabilitation, treatment, and reconstructive surgery and should continue to be followed in outpatient mental health treatment as well. They should be continually screened for exacerbation of symptoms and impairments in functioning. Patients with demoralization, those who develop psychiatric symptoms during the hospital stay, and patients with pre-existing psychiatric diagnoses may be at increased risk for continued psychiatric decompensation and thus should be referred to outpatient mental health follow-up.

The emotional and psychological responses in burn survivors impact many aspects of care and recovery. Highlighting the differential and continuum of symptoms and appropriately screening, treating and providing follow-up care is essential to the care of burn survivors. While we recognize the clinical challenges of differentiating these syndromes as well as gaps in the literature, we recommend utilizing this framework to classify patients. More research is needed to understand the prevalence of these depressive syndromes in burn survivors, and better identify the most appropriate and effective treatment for each syndrome in this population. Being familiar with the continuum and the comorbidity of these distinct clinical entities can help the burn clinician better understand the range of psychological responses to burn injury, identify and characterize symptoms, treat appropriately, and eventually investigate longitudinal outcomes for these patients.

## Figures and Tables

**Table 1:** Differential diagnosis of depressive symptoms in burn survivors.

Demoralization	Adjustment Disorder	Major Depressive Disorder	PTSD/Acute Stress Disorder
Not in DSM-V	Trauma/Stressor related disorder	Mood disorder	Trauma/Stressor related disorder
Frustration, sadness, sense of loss, existential distress	Marked distress, low mood, anxiety, poor sleep	Anhedonia, hopelessness, thoughts of suicide	Intrusive symptoms, avoidance, alteration in mood and arousal
In response to a stressor	In response to a stressor	Does not need to be in response to a stressor	In response to a trauma
Functional impairment not necessary	Functional impairment present	Functional impairment present	Functional impairment present
Mood can brighten with change in circumstance or distraction	Mood is often still reactive to outside circumstances but may not be	Mood is not reactive to outside circumstances; mood is persistently low	Can present with a mood that is not reactive; inability to experience positive emotions

**Table 2:** Management of demoralization, adjustment disorder, and MDD in burn survivors.

Demoralization Management	Adjustment Disorder Treatment	MDD Treatment
Primarily psychotherapy: supportive	Psychotherapy: CBT	Psychotherapy: CBT
Aim to reframe the injury as something patient can overcome or grow from	Learn coping skills to deal with the stressor	Behavioral activation, identify automatic thoughts
Pharmacology as needed for symptomatic problems (poor sleep, low appetite)	Limited evidence for pharmacology Treat symptomatic problems (poor sleep, low appetite, anxiety)	Psychopharmacology Restart home psychiatric medications ECT/rTMS
Any member of the team (doctors, nurses, PT/OT) to provide psychological support	Any member of the team may require psychiatric consultation and follow-up care	Involve psychiatric consultants to ensure safety and follow-up care
